# A statistical approach to assess interspecific consumptive competition and functional redundancy in ephemeral resource uses using camera traps

**DOI:** 10.1002/ece3.70031

**Published:** 2024-07-24

**Authors:** Yoshihiro Nakashima, Akane Hashizume, Akane Kanda

**Affiliations:** ^1^ College of Bioresource Science Nihon University Fujisawa Kanagawa Japan

**Keywords:** Bayesian inference, camera trap, consumptive competition, ephemeral resources, species interactions

## Abstract

Camera traps have been widely used in wildlife research, offering significant potential for monitoring species interactions at ephemeral resources. However, raw data obtained from camera traps often face limitations due to observation censoring, where resource consumption by dominant animals may obscure potential resource use by less dominant animals. We extended time‐to‐detection occupancy modeling to quantify interspecific consumptive competition and redundancy of ecosystem functions through consumption between two species, while accounting for observation censoring. By treating resource use by rival species as censored data, we estimated the proportion of resources potentially used in the absence of rival species and calculated the loss caused by the rival species, which is defined as “Competition Intensity Index.” We also defined the Unique Functional Contribution, which represents the net functional loss when a species is removed, calculated by excluding the contribution potentially substituted by the other species. We also considered resource degradation and computed the quantity of resources acquired by each species. This established framework was applied to predation data on bird nests by alien squirrels and other predators (Case 1) as well as scavenging on mammalian carcasses by two carnivores (Case 2). In Case 1, the introduction of squirrels significantly affected the breeding success of birds. Although nests were being preyed upon by native crows also, our model estimated that Unique Functional Contribution by the squirrels was 0.47. This means that, by eradicating the squirrels, the reproductive success of the birds could potentially increase by as much as 47%. In Case 2, the Competition Intensity Index for foxes was 0.17, whereas that for raccoon dogs was 0.46, suggesting an asymmetric effect of resource competition between the two species. The frequency distribution of wet mass available to the two species differed significantly. This approach will enable a more robust construction of resource–consumer interaction networks.

## Graphical abstract

We extended time‐to‐detection occupancy modeling camera trapping data to quantify interspecific consumptive competition at ephemeral resources and redundancy of ecosystem functions through consumption.

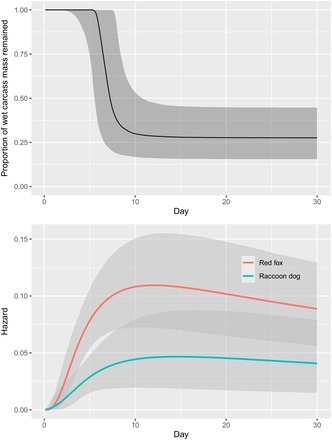

## 1 INTRODUCTION

Camera traps are indispensable tools in wildlife research that offer valuable insights into wildlife ecology, conservation, and management. While widely used to estimate population state variables such as occupancy (Ahumada et al., 2011; Tobler et al., 2015), abundance (Hofmeester et al., 2024; Parsons et al., 2017), and density (Nakashima et al., 2018; Rowcliffe et al., 2008; Royle et al., 2009), the potential of this technique extends beyond population assessment. For instance, camera traps are well suited for monitoring interactions at valuable, ephemeral resources, such as fruits (Yasuda et al., 2005), seeds (Jansen et al., 2012), carcasses (Cunningham et al., 2018; Hashizume et al., 2023), and bird nests (Richardson et al., 2009). In trophic interaction studies, there is a prevalent focus on understanding “what resources specific animals eat,” yet there exists a noticeable gap in data regarding “whom resources are eaten by,” primarily due to methodological constraints (Hoso & Shimatani, 2020). Camera traps offer an opportunity to address this gap and provide valuable insights into the dynamics of food webs and the ecological and evolutionary consequences of trophic interactions (Smith et al., 2020). However, although statistical models exist for estimating population state variables, a comparable framework for comprehending resource utilization is currently lacking. This deficiency results in the suboptimal utilization of the collected information.

Imperfect detection often poses significant challenges in identifying resource users and quantifying the proportion of resources consumed. Imperfect detection refers to situations in which the monitoring of resource use is prematurely censored for various reasons. These may include camera malfunctions or resources that exit the camera frame. This can also occur when multiple species compete for resources. For example, consider a resource crucial for two sympatric species. If one species is more abundant or detects the resource faster than the other species, utilization by the latter, even if the resource is essential for them, may rarely be observed. This indicates a significant potential discrepancy between the frequency of resource use observed by camera traps and the potential frequency of use. Ignoring these unobserved potential resource uses may lead to an underestimation of interspecific consumption competition and the redundancy of ecosystem functions. Furthermore, overlooking such unobserved interactions may contribute to an inaccurate ecological understanding and misguided conservation or management decisions in the face of declining native wildlife or the proliferation of invasive species.

One potential approach to overcome these challenges is to apply a statistical model that has been used to estimate occupancy while accounting for imperfect detection. Specifically, time‐to‐detection (TTD) occupancy modeling (Bornand et al., 2014) may be highly useful, given the similarity between the process of human observers finding wildlife and wildlife detecting resources for the first time. The TTD model extends the parametric survival analysis to allow for the estimation of animal occupancy and detection probability based on a one‐time survey (Bornand et al., 2014). In our context, this model provides a robust means to estimate potential interactions by treating data where animal use was not detected for some reason as “censored data” in survival analysis framework. In cases where resources have been used by other species, considering them as censored data allows for the simultaneous estimation of the proportion of resources that each species could potentially use and the time required for resource detection. This would enable the computation of important metrics related to interspecific interactions using the estimated parameters. For instance, it may be possible to infer the proportion of resources that one species could have used but were preempted by other species, as well as the unique functional contribution (or functional redundancy) of each species through resource consumption. Furthermore, by selecting an appropriate probability distribution to model time‐to‐detection, it may be possible to reveal how detectability (i.e. hazard) changes over time in response to variations in the state of resources.

In this study, we extended time‐to‐detection modeling to quantify interspecific consumptive competition and redundancy of ecosystem functions. Our approach involves placing fresh resources in front of a camera and modeling the time it takes for the resources to be initially visited by animals. Additionally, by separately obtaining data on the state of resources (e.g. nutrients) and modeling their temporal changes, we aimed to estimate how many resources were allocated to which animals. Furthermore, we applied the established framework to predation data on bird nests and the scavenging of animal carcasses. This expanded application allowed us to gain insights into the dynamics of interspecific interactions, providing a comprehensive understanding of resource use patterns and their implications for ecosystem functions.

## 2 METHODS

### 2.1 Model framework

First, let us consider modeling the TTD data (i.e. the elapsed time since placing the fresh resource in front of a camera trap) for a resource exclusive to a particular species *A*. If animals are available at all stations, the observed data ($c_{k}$ and $t_{k}$) at the monitoring station k (k=1,…,K) can be effectively modeled using a survival time analysis framework (Klein & Moeschberger, 2003), where $c_{k}$ indicates whether monitoring is censored (1) or not (0), whereas $t_{k}$ indicates the timing of the visitation by species *A* or that of monitor censoring. Note that “censoring” refers to the situation where the resource use by species *A* was not observed due to any reason, e.g. camera trap malfunction or the resource being utilized by other species. “Available” refers to the state in which animals occupy a site, indicating the potential use of resources by a specific species.

Let us assume that the TTD by species *A* is represented by the probability variable (T) followed by some arbitrary temporal probability distribution (e.g., exponential, gamma distribution), denoted by $f\left(T|\theta^{A}\right)$, where $\theta^{A}$ represents the parameter(s) of the distributions. In this case, if resource use by species *A* is observed at time $t_{k}$ ($c_{k}=1$), the likelihood is simply $f\left(t_{k}|\theta^{A}\right)$. On the other hand, if resource use is not observed until time $t_{k}\left(c_{k}=1\right)$ and monitoring is terminated, the likelihood can be expressed as $S\left(t_{k}|\theta^{A}\right)=1-F\left(t_{k}|\theta^{A}\right)$, where $F\left(T|\theta^{A}\right)$ represent the cumulative probability distribution function of $f\left(T|\theta^{A}\right)$ and defined as $F\left(T|\theta^{A}\right)$ $=\int_{0}^{T}f\left(u|\theta^{A}\right)\mathrm{du}$. In survival analysis framework, $S\left(T|\theta^{A}\right)$ is generally referred to as the survival function and returns the probability that the event did not occur until T. Using these functions, the likelihood of the observed data can be formulated as follows:

(1) $$L\left(\theta^{A}|\left\{t_{1},\ldots ,t_{K},c_{1},\ldots ,c_{K}\right\}\right)=\prod_{k=1}^{K}f{\left(t_{k}|\theta^{A}\right)}^{1-c_{k}}\cdot S{\left(t_{k}|\theta^{A}\right)}^{c_{k}}$$

Note that $c_{k}$ is an indicator that takes the value 1 if the monitoring is censored and 0 otherwise. That is, in the case of censoring $c_{k}=1$, $f{\left(t_{k}|\theta^{A}\right)}^{0}=1$ and the likelihood becomes $S\left(t_{k}|\theta^{A}\right)$. On the other hand, in the case of no censoring $c_{k}=0$, $S{\left(t_{k}|\theta^{A}\right)}^{0}=1$ and the likelihood is $f\left(t_{k}|\theta^{A}\right)$. This formulation allowed us to assess the likelihood of the observed and censored data given the specified distribution for the TTD by species *A*.

In practice, animals are often unoccupied at some stations. Let $\psi^{A}$ denote the occupancy probability of species *A*. In such cases, non‐observations by camera traps may be due to either the station being unoccupied by the species ($1-\psi^{A}$) or monitoring being censored while the species visited the station ($\psi^{A}\cdot S\left(t_{k}|\theta^{A}\right)$). Thus, the likelihood that the data where observation was censored without detection at station k is given by:

(2) $$\begin{array}{c} L\left(\theta^{A},\psi^{A}|\left\{t_{1},\ldots ,t_{K},c_{1},\ldots ,c_{K}\right\}\right)\\ =\prod_{k=1}^{K}{\left\{\psi^{A}\cdot f\left(t_{k}|\theta^{A}\right)\right\}}^{1-c_{k}}\cdot {\left\{\left(1-\psi^{A}\right)+\psi^{A}\cdot S\left(t_{k}|\theta^{A}\right)\right\}}^{c_{k}}\; \end{array}$$

This represents a TTD occupancy model commonly used to estimate animal occupancy considering imperfect detection.

Now, we can extend this model to account for the presence of another species, *B*, which uses the same resources and has a competitive relationship with species *A*. While it is possible to formulate the likelihood by considering the occupancy of both species separately and deriving conditional probabilities for each species, a simpler approach can be adopted by assuming that the detection of the resource is independent for each species. This assumption can be justified for ephemeral resources, in which the availability of resources is unpredictable. We may model the detection times for both species separately, acknowledging that the survey is censored if the rival species uses the resource. The likelihood of the data on species *A* is the same as in Equation 2, and similarly for species *B*. Thus, joint likelihood is given by:

(3) $$\begin{array}{c} L\left(\theta^{A},\theta^{B},\psi^{A},\psi^{B}|\left\{t_{1},\ldots ,t_{K},c_{1}^{A},\ldots ,c_{K}^{A},c_{1}^{B},\ldots ,c_{K}^{B}\right\}\right)\\ =\prod_{k=1}^{K}{\left\{\psi^{A}\cdot f\left(t_{k}|\theta^{A}\right)\right\}}^{1-c_{k}^{A}}\cdot {\left\{\left(1-\psi^{A}\right)+\psi^{A}\cdot S\left(t_{k}|\theta^{A}\right)\right\}}^{c_{k}^{A}}\\ \cdot \prod_{k=1}^{K}{\left\{\psi^{B}\cdot f\left(t_{k}|\theta^{B}\right)\right\}}^{1-c_{k}^{B}}\cdot {\left\{\left(1-\psi^{B}\right)+\psi^{B}\cdot S\left(t_{k}|\theta^{B}\right)\right\}}^{c_{k}^{B}}\; \end{array}$$

where $c_{k}^{A}$ and $c_{k}^{B}$ are an indicator of censoring for species *A* and *B*, respectively, at kth observation. The same approach can also be applied when three or more species compete for resources.

Parameter estimation can be performed through either maximum likelihood or Bayesian estimation, the latter being more convenient because it allows for the construction of prediction distributions (as discussed below). In this modeling approach, a crucial aspect is the selection of the time distribution, $f\left(T|\theta \right)$. The determination of which time distribution provides superior predictive performance can be assessed using relevant indices, such as AIC or WAIC. If animal detection occurs randomly, it follows an exponential distribution. Alternatively, if the detectability (hazard) changes over time, a temporal distribution with an increasing hazard might be more appropriate.

### 2.2 Quantifying ecological metrics

The mentioned approaches allow for the computation of essential ecological metrics. For example, in a system with two species in a competitive relationship, we refer to the proportion of resources potentially available to species *A* but rendered unavailable by the other species *B* as the “Competition Intensity Index” for species *A*. This index can be estimated posteriorly using the inferred parameters the estimated parameters (${\hat{\psi }}^{A},{\hat{\psi }}^{B},{\hat{\theta }}^{A},{\hat{\theta }}^{B})$. That is, species *A* loses resources when species *B* occupies and detects the resources earlier than species *A*. For certain resource types, the state of the resource may suddenly change at a time v since the camera settings and becomes unavailable thereafter (e.g. the departure of nestlings in a bird's nest). Taking such resources into account, the index can be computed as follow:

(4) $$\begin{array}{c} \text{Competition Intensity Index for species}\;A\\ ={\hat{\psi }}^{B}\cdot \int_{0}^{v}F^{B}\left(u|{\hat{\theta }}^{B}\right)\cdot f^{A}\left(u|{\hat{\theta }}^{A}\right)\mathrm{du}\; \end{array}$$

Note that $f\left(T|\theta \right)$ is a probability density function of the TTD and $F\left(T|\theta \right)$ represent its cumulative distribution function. Again, v indicates that the resource is available until that point. If the timing cannot be clearly defined (e.g., when the resource value gradually decreases), it can be set to infinity. For resources whose state changes suddenly, v should be estimated based on field data (e.g., using survival time analysis) or determined from the average value reported in previous literature. In the following definition of the index, we use the notation with v to maintain generality.

It is also possible to separately evaluate the ecological functions of each species. Let us consider species *A* and *B*, both of which play pivotal roles in ecosystem functions through resource consumption, such as seed dispersal or predation. If we consider a system with only species *A*, the proportion of resources consumed by the timing v (i.e., the proportion of ecosystem functions contributed by species *A*, hereafter referred to as the “Potential Functional Contribution” by species *A*) is ${\hat{\psi }}^{A}\cdot F^{A}\left(v|\theta^{A}\right)$. In a system where species *B* is also present, assuming that these species independently use resources, ${\hat{\psi }}^{A}\cdot F^{A}\left(v|\theta^{A}\right)\cdot {\hat{\psi }}^{B}\cdot F^{B}\left(v|\theta^{B}\right)$ of the resources can potentially be used by both species. This value can be regarded as functional redundancy. The distinctive functionality of species *A* (referred to as its “Unique Functional Contribution”), obtained by excluding functions that species *B* can substitute, is expressed as.

(5) $$\begin{array}{c} \text{Unique Functional Contribution}\;\mathrm{by}\;\text{species}\;A\\ ={\hat{\psi }}^{A}\cdot F^{A}\left(v|\theta^{A}\right)-{\hat{\psi }}^{A}\cdot F^{A}\left(v|\theta^{A}\right)\cdot {\hat{\psi }}^{B}\cdot F^{B}\left(v|\theta^{B}\right)\; \end{array}$$

This also applies to regular resources without sudden state changes, where v is set to infinity. The Unique Functional Contribution Index can be interpreted as a net loss of functionality resulting from the absence of species *A*. If the unique functional contribution of species *A* is 25%, it suggests that if species *A* is removed from the ecosystem, 25% of the resources cannot be consumed by other species, and consequently, the ecosystem functions associated with their consumption would be lost. Where species *A* has a negative impact as an invasive species, this represents the net impact that is mitigated by its eradication (see below).

Furthermore, it is possible to compute the expected value regarding the allocation of nutrition within the resources. The nutritional value of ephemeral resources (e.g. carcasses and fruits) may decrease rapidly over time. In this case, the expected value of the resource quantity that species *A* can utilize is the proportion of resources it can discover multiplied by the remaining resource value at the time of first detection (which indicates how much of a given resource is still present). If the proportion of the resource value remaining at time t is denoted as $g\left(t\right)$, the resources that species *A* can effectively acquire can be determined using the following expression:

(6) $$\begin{array}{c} \text{Resource allocation to species}\;A\\ =\int_{0}^{v}{\hat{\psi }}^{A}\cdot \left\{\left(1-{\hat{\psi }}^{B}\right)+{\hat{\psi }}^{B}\cdot S^{B}\left(u|{\hat{\theta }}^{B}\right)\right\}\cdot f^{A}\left(u|{\hat{\theta }}^{A}\right)\cdot g\left(u\right)\mathrm{du}\; \end{array}$$

$g\left(t\right)$ should be determined based on monitoring data for a metric representing resource value (e.g., weight or specific nutritional content). The functional form used to represent the temporal change in resource value is situation‐dependent, but a simple and interpretable function, such as an exponential or Gompertz function, is preferable. By comparing the estimated $g\left(t\right)$ with the hazard of the time distribution from the TTD occupancy model selected as the best predictive model, it may be possible to reveal how detectability (i.e. hazard) changes over time in response to variations in the state of resources. The frequency distribution of acquired nutritional values may be of greater interest than expected values. In this case, constructing a posterior predictive distribution using Bayesian parameter estimation can be beneficial, as illustrated below.

### 2.3 Case study

#### 2.3.1 Case 1: Quantifying the net impacts of nest predation by an invasive species

We applied this approach to actual data to quantify the net impact of nest predation by invasive Formosan squirrels, *Callosciurus erythraeus*, in southeastern Kanagawa Prefecture, Japan, where their population have been increasing. These squirrels frequently prey on bird nests, raising concerns about their impact on native avian breeding (Tamura & Yasuda, 2023). However, there are native nest predators such as large‐billed crows (*Corvus macrorhynchos*), and even if the squirrels were eradicated, the predation pressure on nests might not change. To evaluate the actual effect, we need to evaluate the net impact of nest predation (i.e., the Unique Functional Contribution Index) by excluding the proportion of nests predated by native species.

We conducted artificial nest experiments in an evergreen forest in the Futagoyama region. The characteristics of the nests, including shape, size, and nesting location, were modeled after those of the warbler's nest. We modified commercially available bowl‐shaped nests into cup‐shaped nests (approximately 10 cm in diameter and 6 cm in depth) following Seki and Yasuda (2018). The nests were installed at a minimum distance of 300 m from each other, and placed at a height of approximately 2 m in a tree. Each nest was equipped with two quail eggs and monitored using camera traps (Browning Strike Force, Browning, Morgan, Utah, USA).

The survey was conducted between April and July, 2020. In each survey, the nests were set up at 7–10 locations. The survey was repeated five times, resulting in 32 total nest locations. For subsequent surveys (from the 2nd survey onwards), the nests were placed at different positions within 30 m of the locations of the nests in the previous survey.

We used a TTD occupancy model to analyze the duration until the egg was initially preyed upon. Considering the hatching period of warbler nestlings, we calculated the expected proportion of nests preyed upon within a 14‐day window using Equation 5. We assumed that the resource value remained constant over the 14‐day period. Parameter estimation was conducted using a probabilistic programming language for Bayesian inference, Stan 2.34.1 (Carpenter et al., 2017) with R 4.3.3 language (R Core Team, 2022). Considering the limited sample size and experimental conditions, we fitted an exponential distribution for the TTD data without any fixed and random effects. We specified non‐informative or weakly informative priors for all parameters and ran five chains for each model with 5000 iterations, including a warm‐up period of 2000 iterations. Convergence of the sampling was confirmed, ensuring an R‐hat value (Gelman et al., 2013) of less than 1.1. We reported the median and 95% credible interval (95% CI) of parameters or median and 95% prediction interval based on the posterior sample, unless explicitly stated otherwise. The code is accessible from a data repository (Nakashima et al., 2024).

#### 2.3.2 Case 2: Quantifying the carcass masses obtained by two carnivore scavengers

We modeled the detection of raccoon carcasses using two carnivore scavengers (red foxes, *Vulpes vulpes*, and raccoon dogs, *Nyctereutes procyonoides*) in the Yakumo Study Forest in Hokkaido, Japan. Time‐to‐detection data from Hashizume et al. (2023) were used. The aim of this model is to quantify the Competition Intensity Index (Equation 4) and Unique Functional Contribution Index (Equation 5). We also predicted the resource allocation (Equation 6) for each carnivore species, considering temporal changes in resource availability. Unlike eggs within a nest, carcasses do not undergo a sudden change in resource state, so v was set to infinity.

Between 2016 and 2019, 12–23 raccoon carcasses (69 in total) were placed at least 500 m apart and monitored using camera traps. Additionally, we visited 2–10 carcasses (a total of 21) daily and measured their wet masses (this survey was not conducted in 2016). For detailed information, please refer to Hashizume et al. (2023). The wet mass of carcasses, influenced by maggot activity, rapidly decreased and reached equilibrium (Figure S1). We therefore assumed that the wet mass loss followed a Gompertz function with a gamma distribution error. We parameterized the proportion of expected wet mass $g\left(t\right)$ remaining at time t:

(7) $$g\left(t\right)=1+\left\{1-\left(1-\gamma \right)\cdot \exp\;\left(-\exp\left(-\beta \times \left(t-\alpha \right)\right)\right)\right\}$$

Here, β represents the slope at the inflexion point, α indicates its timing, and γ represents the proportion of the initial mass reaching the equilibrium state.

We conducted a Bayesian estimation for both the TTD model and Gompertz regression using Stan. Due to the limited sample size, we pooled data from a 4‐year period. Although foxes and raccoon dogs did not eat during their initial visits (Hashizume et al., 2023), we modeled the timing of their first visit to quantify the resources potentially available to them. One carcass was visited by a large‐billed crow (*Corvus macrorhynchos*) before a fox and raccoon dog. The data were censored for both species. We obtained the posterior predictive distributions for competition intensity index (Equation 3) and resource allocation (Equation 4) based on the posterior samples. For resource allocation, values exceeding one were adjusted to one in the predictive distribution. Non‐informative or weakly informative priors were used for all parameters. We assumed that TTD followed four candidate distributions (exponential, gamma, log‐normal, and Weibull) and selected the log‐normal distribution as the most predictive distribution based on the WAIC values (Hashizume et al., 2023). For the time distribution, a more intuitive distribution such as an exponential, Weibull, or gamma distribution is preferable, assuming a constant or monotonically changing hazard. However, since carcasses may temporarily become more detectable during the peak of decomposition, we also included the log‐normal distribution. The Stan code is accessible from a Data Repository (Nakashima et al., 2024).

## 3 RESULTS

### 3.1 Case study 1

A total of 32 nests were successfully monitored using camera traps. Among these, 15 nests (46.9%) were observed with the Formosan squirrels preying on eggs, and five nests (15.6%) were preyed upon by native large‐billed crows. The remaining nests (12; 37.5%) showed no signs of predation. The estimated parameter values of the TTD occupancy model are shown in Table 1. The Potential Functional Contribution by the squirrels (i.e., the expected proportion of nests preyed upon by squirrels in the absence of crows) was 0.51 (95% credible interval: 0.36–0.65), while that by crows was 0.25 (0.12–0.41) of the nests, respectively. The Unique Functional Contribution by the squirrels (the proportion of predation contributed solely by squirrels, considering predation by crows) was estimated to be 0.47 (0.27–0.65), whereas that of the crows was 0.18 (0.07–0.43). This means that if the squirrels were eradicated, considering the predation by crows, approximately half of the nests would be spared from predation.

**TABLE 1:** Results of analyzing predation on 32 artificial nests by two predators (Formosan squirrels, *Callosciurus erythraeus* and large‐billed crows, *Corvus macrorhynchos*) between April and July, 2020 in Kanagawa Prefecture, Japan, using a time‐to‐detection occupancy model with an exponential distribution defined by the parameter λ.

Species	*N* ^a^	$\hat{\psi }$		$\hat{\lambda }$		Potential functional contribution		Unique functional contribution	
Formosan squirrel	15	0.52	(0.36–0.66)	0.39	(0.23–0.60)	0.51	(0.36–0.65)	0.47	(0.27–0.65)
Large‐billed crows	5	0.28	(0.13–0.65)	0.21	(0.04–0.50)	0.25	(0.12–0.41)	0.18	(0.05–0.43)

*Note*: The median and 95% credible intervals of the posterior samples were shown.

^a^ Number of nests preyed upon by each species.

### 3.2 Case study 2

**FIGURE 1:**
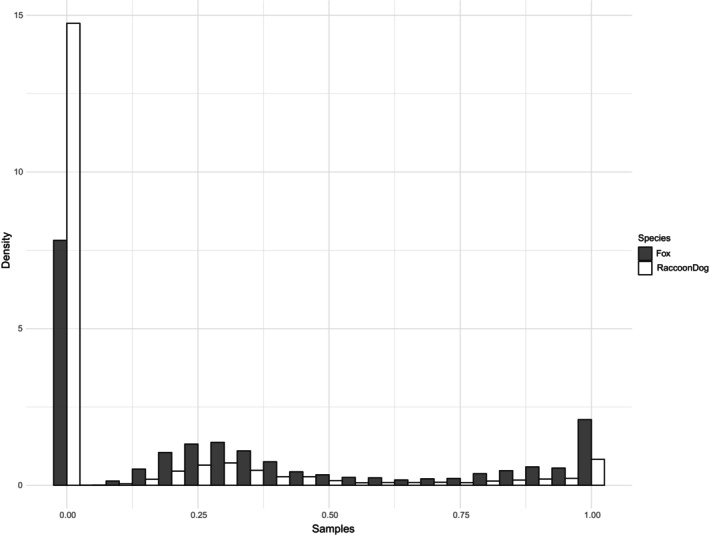
Expected temporal changes in wet mass of raccoon carcasses (upper panel) and their detectability (i.e. hazard) by red foxes (*Vulpes vulpes*) and raccoon dogs (*Nyctereutes procyonoides*) (lower panel) during the summer in Yakumo Forest, Hokkaido, Japan. The light gray shading represents the 95% credible intervals, with those of wet mass depicting those of the expected values for each carcass.

A total of 69 carcasses were monitored using camera traps (Table 2). Among the placed carcasses, 37 (53.6%) were first detected by foxes and 15 (21.7%) by raccoon dogs. The detectability of resources (hazard) increased until ca. 10 days and then decreased gradually (Figure 1). The estimated parameter values of the TTD occupancy model are shown in Table 2. The TTD model estimated that the first visitation occurred at 9.15 days (95% prediction interval: 2.6–34.4) for red foxes and at 6.8 days (3.4–131.0) for raccoon dogs. Red foxes and raccoon dogs indeed competed for raccoon carcasses. The Competition Intensity for red foxes (i.e., the proportion of potentially available resources rendered unavailable due to the presence of raccoon dogs) was 0.17 (0.11–0.26), and that for raccoon dogs was 0.46 (0.33–0.62). In other words, red foxes had a competitive advantage over raccoon dogs. Consequently, there was a substantial difference in their Unique Functional Contributions, with red foxes at 0.38 (0.12–0.54) and raccoon dogs at 0.12 (0.06–0.24).

**TABLE 2:** Results of analyzing scavenging on 69 carcasses by two carnivore species (red foxes, *Vulpes vulpes*, and raccoon dogs, *Nyctereutes procyonoides*) in Hokkaido, Japan, using a time‐to‐detection occupancy model with a lognormal distribution defined by the parameters μ and σ.

Species	*N* ^a^	$\hat{\psi }$		$\hat{\mu }$		$\hat{\sigma }$		Competition intensity		Unique functional contribution	
Red fox	37	0.74	(0.61–0.86)	2.16	(1.99–2.38)	0.64	(0.53–0.83)	0.17	(0.11–0.26)	0.38	(0.12–0.54)
Raccoon dog	15	0.48	(0.30–0.83)	2.48	(2.10–3.41)	0.72	(0.49–1.35)	0.46	(0.33–0.62)	0.12	(0.06–0.24)

*Note*: The median and 95% credible intervals of the posterior samples are shown. In this case, the values of $\hat{\psi }$ represent the potential functional contribution.

^a^ Number of carcasses detected by each species.

**FIGURE 2:**
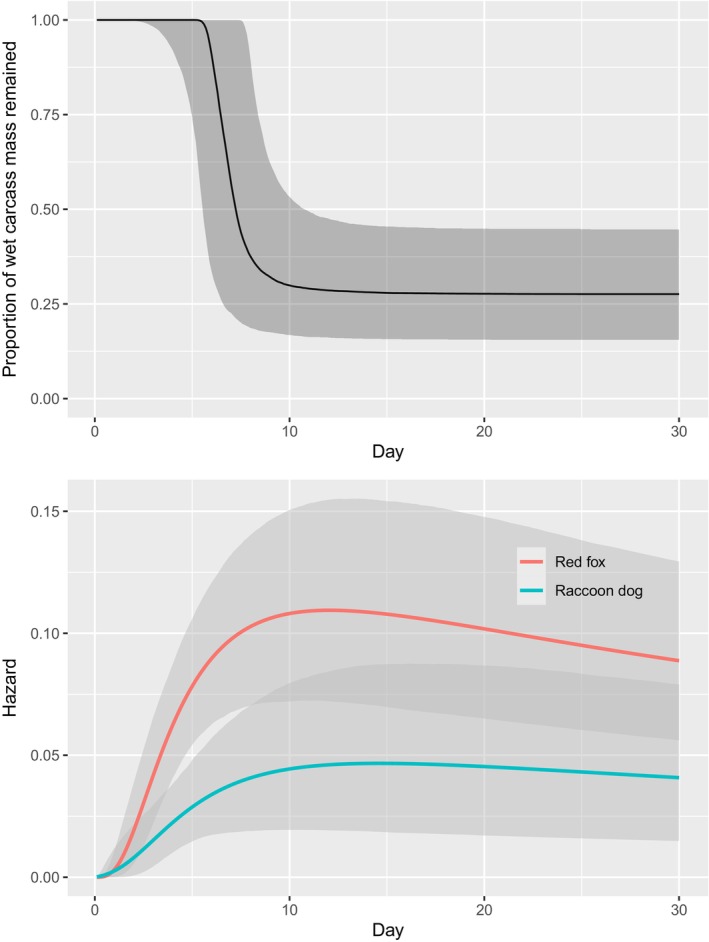
The remaining wet carcass mass when each of the two scavengers (red foxes and raccoon dogs) initially visited a carcass during the summers of 2016–2019 in Yakumo study forest, Hokkaido, Japan. In cases where the carcass was visited first by a rival scavenger or the species did not occur, the value is 0. If visited after the mass had decreased primarily due to maggot activity, the value falls between 0 and 1. For more details, refer to the main text.

The wet mass of resources (resource allocation) acquired by red foxes was much larger than that acquired by raccoon dogs. The posterior predictive distribution of the weight allocation available to foxes and raccoon dogs is shown in Figure 2. In this histogram, zero indicates cases where it was taken by a rival species or the species did not occur, whereas one indicates cases where scavengers detected carcasses before the carcass mass decreased due to maggot activity. Values between zero and one represent cases in which carcasses were discovered before a rapid decrease in quality occurred and before being detected by rival scavengers.

## 4 DISCUSSION

We developed a statistical framework that models TTD data to quantify the intensity of resource competition and functional redundancy in ephemeral resource use between the two species. Various ephemeral resources have been monitored in numerous studies using camera traps. However, many of these studies have used a simpler approach (e.g. Mayfield method, Mayfield, 1961), or semiparametric models such as the proportional hazard model (Jensen et al., 2023; Kitamura et al., 2008). Our parametric modeling approach enables the computation of ecologically important metrics that would otherwise be challenging to derive. Our approach, which can be easily applied to previously acquired data, serves as an effective method for quantitatively evaluating interspecies interactions in ephemeral resources.

Our empirical data analysis has intriguing implications for ecology and conservation. In Case 1, we demonstrated that the introduction of squirrels significantly affected the breeding success of native birds. The model estimated that the breeding success was enhanced by 47% by the removal of the squirrels. This assumes that predator abundance and behavior remains unchanged after eradication. Moreover, nest experiments using artificial nests may overestimate predation pressure (Richardson et al., 2009). Nonetheless, these estimates provide compelling evidence that underscores the urgent need for eradication by effectively communicating this need to both authorities and the public. While previous studies have compared nest predation before and after the eradication of invasive species (Jones et al., 2005), our approach is preferable for verifying the actual effects of invasive species because it allows us to estimate the effects of eradication in advance. This can provide a basis for determining how to allocate limited human and financial resources.

In Case 2, we observed a pronounced, asymmetric impact of resource competition between two carnivore scavengers, red foxes and raccoon dogs. Specifically, raccoon dogs, a competitively inferior species, exhibited 46% loss of potentially usable carcasses (Competition Intensity Index for red foxes = 0.46, Table 2) when red foxes, the dominant species, were present. On the other hand, the proportion lost by red foxes due to the presence of raccoon dogs was only 17% (Competition Intensity Index = 0.17). Consequently, the frequency distribution of the quantity of resources available to the two species differed significantly (Figure 2). Our observations included not only the impact of rival scavengers but also a decrease in the remaining wet mass due to maggot activity. Interestingly, the hazard of the TTD occupancy model increased with the decrease in carcass weight, suggesting that the two species utilized the volatile compounds generated during this process as cues to detect the carcasses. Although the wet mass of the monitored carcasses may not necessarily correlate with nutrient content, a more detailed measurement of changes in the nutritional values of carcasses could facilitate a quantitative comparison of resource competition with taxonomically distant species, such as maggots, enabling the assessment of the relative importance of competition with closely related species. In addition, reflecting the differences in the time taken to detect carcasses, there were also differences in the Unique Functional Contributions of red foxes and raccoon dogs. Conventional studies often assume that the relative importance of a species' function corresponds to the observed proportion (Nakashima et al., 2010), which inevitably leads to an underestimation of redundancy. Therefore, our approach would be effective in evaluating the robustness of interaction networks and assessing their nestedness.

The appropriate application of our model requires an understanding of its constraints, especially the need for ample TTD data. In scenarios involving multiple resource users, the sample size for each species inevitably diminishes compared with scenarios involving a single species. Competitively inferior species are particularly likely to have limited sample sizes. In such cases, it is necessary to devise measures, such as establishing processing zones with restricted access to dominant species. Furthermore, this paper has assumed the application of the TTD occupancy model for a single‐visit survey. However, by taking temporal repetitions, it is possible to apply the model for multiple visits surveys, which would significantly improve the efficiency of the model. However, in this case, it is necessary to pay close attention to the location and timing of resource placement to ensure independent resources between sampling occasions.

Furthermore, defining the scope of the model is crucial. In our model, we assumed that the ephemeral resources were exhausted during a single visit. It is essential to model nonindependent visit timings when resources can be used multiple times. Consider larger carcasses, for instance, where the initial use by large carnivores enables smaller species to subsequently use them (Naves‐Alegre et al., 2024). Conversely, large species can directly (i.e. inference competition) or indirectly (fear effect) influence the consumption of food by smaller species (Allen et al., 2015; Cunningham et al., 2018). The timing of multiple visits by these animals can be modeled using an inhomogeneous point process (e.g. a multivariate Hawkes process) that allows more complex direct and indirect species interactions to be quantified. Creating models capable of effectively utilizing the valuable data provided by camera traps, which capture timed detection, represents a future research challenge in evaluating these complex interactions.

In this study, we introduced a framework for modeling TTD data gathered from camera traps used to monitor ephemeral resources. Our approach enables the computation of ecologically significant metrics such as resource competition intensity and functional redundancy. The model used in this study is based on the TTD occupancy model, a method previously used in individual population surveys which offers simplicity in understanding and implementation. Although the probability distribution is restricted to exponential and Weibull distributions, the model can be run using the “unmarked” package (Fiske & Chandler, 2011) in R. Furthermore, we provide a Stan code for parameter estimation based on a Bayesian framework. By using standardized analysis methods, comparisons between sites become more straightforward and the potential for conducting reliable meta‐analyses is enhanced. We anticipate the widespread utilization of this framework in various locations in the future.

## AUTHOR CONTRIBUTIONS

**Yoshihiro Nakashima:** Conceptualization (lead); funding acquisition (lead); methodology (lead); project administration (lead); software (lead); supervision (lead); visualization (lead); writing – original draft (lead). **Akane Hashizume:** Data curation (equal); formal analysis (supporting); investigation (lead); writing – original draft (supporting); writing – review and editing (equal). **Akane Kanda:** Data curation (equal); formal analysis (equal); investigation (equal); validation (equal); writing – original draft (equal); writing – review and editing (equal).

## CONFLICT OF INTEREST STATEMENT

The authors have no conflict of interest to declare.

## STATEMENT ON INCLUSION

All authors were engaged early in the research and study design process to ensure that the diverse sets of perspectives they represented were considered from the outset. Whenever relevant, literature published by scientists from the region was cited and efforts were made to consider relevant works published in the local language.

## Supporting information


Figure S1.

## Data Availability

The data and code for the manuscript are available from the Dryad Digital Repository https://doi.org/10.5061/dryad.kkwh70sbn.
