# Supplementary material for: A statistical approach to assess interspecific consumptive competition and functional redundancy in ephemeral resource uses using camera traps
Source: Ecol Evol. 2024 Jul 24;14(7):e70031. doi: 10.1002/ece3.70031 (PMC11268935; doi:10.1002/ece3.70031)
Supplement: Supplementary file 1 — Figure S1. [file ECE3-14-e70031-s001.docx]

# **Supporting Information**


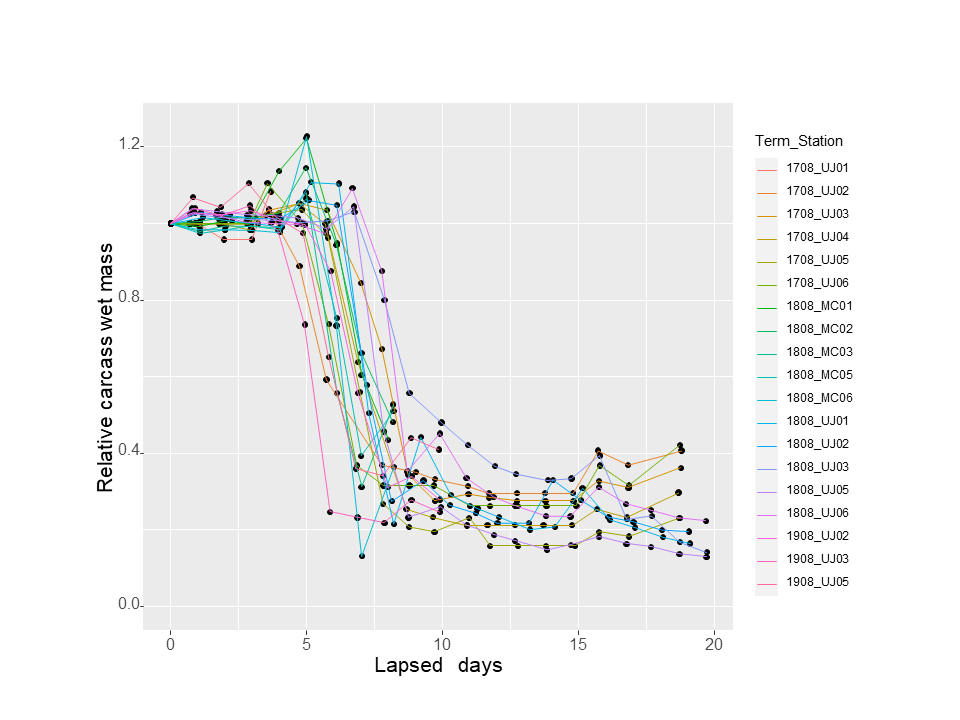


Figure S1. Temporal loss of wet mass (%) of raccoon carcasses in the summer of 2017–2019 in Yakumo forest, Hokkaido, Japan. The decline in wet mass exhibited sigmoidal patterns.
